# The association of allergic asthma and carotid intima-media thickness in adolescence: data of the prospective early vascular ageing (EVA)-Tyrol cohort study

**DOI:** 10.1186/s12872-021-02452-1

**Published:** 2022-01-18

**Authors:** Bernhard Winder, Sophia Zollner-Kiechl, Nadja M. Gruber, Benoît Bernar, Nina Gande, Anna Staudt, Katharina Stock, Christoph Hochmayr, Ralf Geiger, Andrea Griesmacher, Markus Anliker, Stefan Kiechl, Ursula Kiechl-Kohlendorfer, Michael Knoflach, the Early Vascular Ageing (EVA)-Tyrol Study Group, Carmen Reiter, Christina Schreiner, Julia Klingenschmid, Julia Marxer, Martina Kothmayer, Maximilian Pircher, Manuela Bock-Bartl, Mandy Asare, Maximilian Bohl, Raimund Pechlaner

**Affiliations:** 1 Department of Pediatrics II, Medical University of Innsbruck, Anichstrasse 35, 6020 Innsbruck, Austria; 2 VASCage, Research Centre on Vascular Ageing and Stroke, Innrain 66a, 6020 Innsbruck, Austria; 3 Department of Neurology, Medical University of Innsbruck, Anichstrasse 35, 6020 Innsbruck, Austria; 4 Department of Pediatrics I, Medical University of Innsbruck, Anichstrasse 35, 6020 Innsbruck, Austria; 5 Department of Pediatrics III, Medical University of Innsbruck, Anichstrasse 35, 6020 Innsbruck, Austria; 6 Central Institute of Clinical Chemistry and Laboratory Medicine (ZIMCL), Medical University of Innsbruck, Anichstrasse 35, 6020 Innsbruck, Austria

**Keywords:** Atherosclerosis, Allergic asthma, Clinical studies, Carotid intima-media thickness

## Abstract

**Background:**

In recent years, there has been increasing evidence that asthma is associated with atherosclerosis and cardiovascular disease. However, data in children and adolescents are scarce and conflicting. We aimed to assess the impact of asthma with and without an allergic component on the carotid intima-media thickness in a large pediatric population.

**Methods:**

The community-based early vascular ageing-Tyrol cohort study was performed between May 2015 and July 2018 in North, East (Austria) and South Tyrol (Italy) and recruited youngster aged 14 years and above. Medical examinations included anthropometric measurements, fasting blood analysis, measurement of the carotid intima-media thickness by high-resolution ultrasound, and a physician guided interview.

**Results:**

The mean age of the 1506 participants was 17.8 years (standard deviation 0.90). 851 (56.5%) participants were female. 22 subjects had a physician diagnosis of non-allergic asthma, 268 had inhalative allergies confirmed by a positive radio-allergo-sorbent-test and/or prick test, and 58 had allergic asthma. Compared to healthy controls, participants with non-allergic asthma (411.7 vs. 411.7 µm; *p* = 0.932) or inhalative allergy (420.0 vs. 411.7 µm; *p* = 0.118) did not have significantly higher carotid intima-media thickness (cIMT). However, participants with allergic asthma had significantly higher cIMT (430.8 vs. 411.7; *p* = 0.004) compared to those without and this association remained significant after multivariable adjustment for established cardiovascular risk factors.

**Conclusion:**

Allergic asthma in the youth is associated with an increased carotid intima-media thickness. Physicians should therefore be aware of allergic asthma as a potential cardiovascular risk factor in children and adolescents.

*Trial Registration Number* The EVA-Tyrol Study has been retrospectively registered at clinicaltrials.gov under NCT03929692 since April 29, 2019.

## Background

Asthma and cardiovascular disease (CVD) are highly prevalent conditions worldwide [1, 2]. In recent years, evidence has accumulated that asthma is associated with CVD [3–10]. However, available epidemiological and clinical data are not univocal in supporting this hypothesis [11, 12], and pathophysiological links between these two diseases remain poorly defined. Given the important role of inflammatory processes and the immune system in human atherogenesis [13, 14], it is plausible that subjects suffering from chronic inflammation—a hallmark characteristic of asthma—are more prone to CVD [15]. In addition, asthma has been associated with several cardiovascular risk factors (CVRF) such as elevated body-mass index (BMI) [16, 17] and biomarkers that were also associated with CVD like high levels of tumor necrosis factor-α [18, 19], Interleukin 6 [20], and fibrinogen [5, 21].

Considering the recent estimated prevalence of 8.3% for asthma in children and adolescents in the United States [22], asthma is a major health burden. Although atherosclerosis may originate in childhood [23], data on atherosclerotic vascular changes in children and adolescents with asthma are scarce and conflicting [24–28].

With data of the Atherosclerosis Risk Factors in Male Youngsters (ARMY) study (participants aged 17 and 18 years) and the Bruneck study (men and women aged 40–70 years), we have previously demonstrated a significant association between atherosclerosis and the common allergic diseases asthma and allergic rhinitis [24]. However, differentiation between asthma with and without an allergic component was not possible due to the low number of asthmatics in these populations.

The purpose of the present study was to investigate the impact of asthma with and without an allergic component on carotid intima-media thickness (cIMT), a marker of early subclinical atherosclerosis [29] and predictor for future vascular events [30], in the early vascular ageing (EVA)-Tyrol cohort of healthy young adolescents.

## Methods

Data that support the findings of this study are available from the corresponding author upon request.

### Study design and participants

The EVA-Tyrol cohort study was a community-based non-randomized, controlled trial performed between May 2015 and July 2018 in North, East (Austria) and South Tyrol (Italy) aiming to assess the efficacy of a health promotion intervention on CVRF, behaviors as well as on vascular wall changes in healthy adolescents. The study protocol including detailed methods has been published [31]. In brief, students attending 9th or 10th grade (target age 14–16 years) and apprentices of the same age were invited to participate. Baseline examinations were performed between May 2015 and December 2016. After approximately two years (target age 16–18 years), follow-up was performed between August 2017 and July 2018. Simultaneously, another group of adolescents took part as participants in the control group. To achieve a representative sample of adolescents homogenous in age, participants of the intervention group as well as the control group were included in the present exploratory analysis.

The study was approved by the local ethics committee of the Medical University of Innsbruck (approval number AN 2015-0005 345/4.13) and was executed in agreement with the Declaration of Helsinki. The trial has been registered at clinicaltrials.gov (NCT03929692). All participants provided a written informed consent or if the participants had not attained age of majority, the consent was additionally provided by a parent or legal guardian.

### Diagnosis of asthma and allergy

The medical history of allergies and asthma was assessed in a physician guided, face-to-face interview. Consistent with other studies [4, 12] and the previous finding that, in the absence of pulmonary function testing, self-reported questions about physician diagnosed asthma have the highest diagnostic value for asthma diagnosis [32], we defined asthma as prevalent if the participants either answered positive to the question: "Has a doctor ever told you that you have asthma?” and/or the participant reported an ongoing anti-asthmatic drug therapy, both on a regular basis or on demand. The presence of an allergy was defined if an inhaled allergen was self-reported to cause repeated clinical symptoms and the diagnosis was confirmed by a positive radio-allergo-sorbent-test (RAST) or prick test. Allergic asthma was recorded if both asthma and allergy were present.

### Anthropometry

For measurement of height with a Harpenden stadiometer (Holtain, Crymych, United Kingdom) and weight with calibrated medical precision scales, participants were clothed with light indoor clothes without shoes. The calculation of BMI was done by dividing the body weight in kilograms by the square of height in meters. According to the recommendations of the WHO [33] waist circumference was measured with a stretch-resistant tape to the nearest 0.1 cm. After 5 min of rest systolic and diastolic blood pressure were calculated as the mean of 3 independent measurements on the left and right upper arm in a sitting position (automated oscillometric device OMRON M4-I, Omron Healthcare Co., Lake Forest, Illinois, USA).

### Assessment of lifestyle risk factors

We assessed behavioral risk factors by standardized medical interviews with questionnaires adapted from the Atherosclerosis Risk Factors in Female Youngsters (ARFY), Atherosclerosis Risk Factors in Male Youngsters (AMRY) and Bruneck studies [34–36]. Smoking and physical activity were assessed in physician-guided interviews. Cigarette pack years were calculated by multiplying the number of packs of cigarettes smoked per day by the number of years the person has smoked. The average number of minutes per day of moderate- or vigorous exercise (i.e., leading to an increased heart rate and/or sweating) served as metric for physical activity.

### Laboratory methods

Overnight fasting blood samples were cooled and delivered to the Central Institute for Medical and Chemical Laboratory Diagnosis of the Medical University of Innsbruck, Austria immediately. A standard enzymatic colorimetric assay (Cobas 8000, Roche Diagnostics, Rotkreuz, Switzerland) was used to measure total cholesterol, high-density lipoprotein-cholesterol (HDL-C), low-density lipoprotein-cholesterol (LDL-C), and triglyceride. According to the recommendations of the international federation of Clinical Chemistry and Laboratory Medicine we measured Alanine aminotransferase (ALT) (Cobas 8000, Roche Diagnostics, Rotkreuz, Switzerland). Measurement of C-reactive protein (CRP) was conducted with a particle-enhanced immunological clouding assay (Cobas 8000, Roche Diagnostics, Rotkreuz, Switzerland).

### High-resolution ultrasound

Intima-media thickness was measured on the far-wall of the common carotid arteries (cIMT) visualized by high-resolution ultrasound (6.0–13.0 MHz linear probe, GE 12L-RS, on a Vivid q ultrasound device,both General Electric Healthcare, Chicago, Illinois, USA). Three representative measurements in longitudinal images on the distal 4 cm on both sides were done on digitally stored images by a single rater, experienced in ultrasound techniques without information on clinical characteristics of the participant. The mean from all six measurements was used for the present analysis (cIMT_MEAN_).

### Statistical analysis

For statistical analysis we used SPSS version 27.0 (IBM Corporation, Armonk, New York, USA) and R version 4.0.3 (R Foundation for Statistical Computing, Vienna, Austria). Our primary outcome parameter was cIMT_MEAN_. Participants without valid cIMT measurements were excluded from our analysis. Characteristics of the study cohort are presented as number (percentage), mean ± standard deviation or median (Q_25_–Q_75_). Power analysis indicates that, given standard deviations of cIMT_MEAN_ within and sample sizes of subgroups (allergic asthma, controls), we were powered to detect a difference in cIMTmean between groups of at least 19.7 µm, at an alpha level of 0.05, with power of 0.8. Univariate analysis was performed using the Welch-t-test and the Chi-squared test. For multivariable linear regression analysis variables known to impact cIMT (dependent variable) in our study cohort (age, sex, size, systolic blood pressure, physical activity, cigarette pack years, LDL-C, ALT) [37] as well as other common CVRF (waist circumference, CRP)were included as independent variables/confounders into the model. In addition, subgroups (non-allergic asthma, inhalative allergy, allergic asthma) and controls (free of any asthma and inhalative allery) were separately included as dichotomous independent variable. Variables not meeting the assumptions of a normal distribution were log_e_-transformed. All models were inspected for collinearity by variance inflation factors (VIF). Subsequently, the conditions of linearity of the relationship between dependent and independent variables, as well as homoscedasticity, independence and normality of the errors were tested and satisfied. Hence, a multiple linear regression model was finally calculated to assess the impact of the independent variables/confounders on the cIMT_MEAN_ as the primary outcome parameter. *p* Values < 0.05 were considered as statistically significant.

## Results

**Fig. 1:**
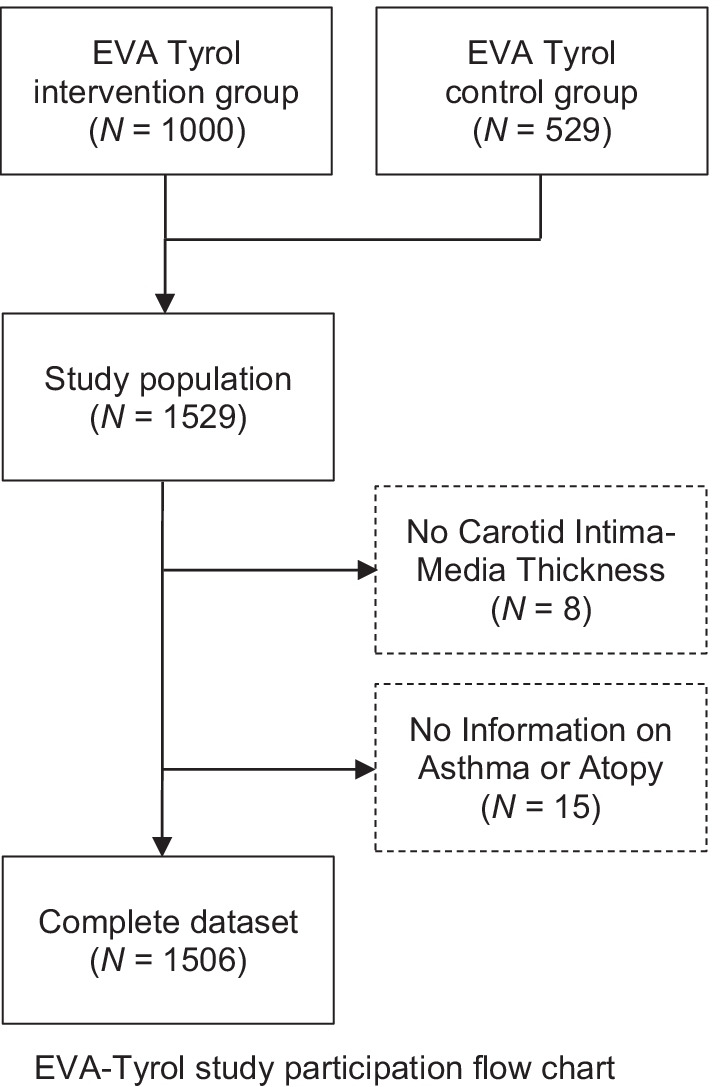
From: The association of allergic asthma and carotid intima-media thickness in adolescence–Data of the prospective early vascular ageing (EVA)-Tyrol cohort study

A flow chart of the study population is shown in Fig. 1. Of the 1529 subjects included in the present analysis, 1000 (65.4%) were in the intervention group and 529 were in the control group. Ultrasound measurement of cIMT was available in 1521 (99.5%) and information on asthma and allergy in 1514 (99.0%) participants leaving 1506 (98.5%) with a complete dataset for the present analysis. The mean age of the participants at the time of examination was 17.8 years (Standard deviation 0.9, range 16–23 years). 851 (56.5%) participants were female. Median cIMT_MEAN_ was 413.3 µm (381.7–446.7) in the whole study population (males: 426.7 µm (395.0–461.7), females: 405.0 µm (375.0–436.7); univariable *p* < 0.001). Further characteristics of the study population are shown in Table 1.

**Table 1:** Characteristics of the study population

	All	Subjects with allergic asthma	Subjects without any asthma or inhalative allergies	*p* Value
	*N* = 1506	*N* = 58	*N* = 1158	
*Demographics*				
Age (years)	17.8 ± 0.9	17.6 ± 0.8	17.8 ± 0.9	0.156
Sex				
Male	655 (43.5%)	34 (58.6%)	463 (40.0%)	
Female	851 (56.5%)	24 (41.4%)	695 (60.0%)	0.005
*Anthropometrics*				
Size (cm)	172.0 (166.0–179.0)	173.3 (166.3–179.6)	171.5 (166.0–179.0)	0.380
Weight (kg)	64.7 (57.8–73.7)	67.3 (60.3–75.3)	64.3 (57.1–73.5)	0.069
BMI (kg/m^2^)	21.7 (19.8–24.1)	22.1 (20.2–24.9)	21.6 (19.8–24.0)	0.088
Waist circumference (cm)	73.5 (66.5–80.3)	73.0 (64.6–82.8)	73.0 (66.5–80.0)	0.430
*Physical activity*				
Physical activity (min/day)	38.8 (20.3–60.0)	60.0 (33.8–75.3)	34.0 (20.0–60.0)	0.010
*Hemodynamics*				
Systolic blood pressure (mmHg)	121.0 (113.0–130.3)	126.3 (118.3–133.1)	120.3 (112.3–129.7)	0.002
Diastolic blood pressure (mmHg)	70.3 (65.0–76.0)	71.0 (65.0–78.3)	70.3 (65.0–76.0)	0.297
*Smoking*				
Current Smoker	410 (27.2%)	20 (34.5%)	295 (25.5%)	0.128
Ever Smoker	632 (42.3%)	26 (44.8%)	476 (41.1%)	0.612
Pack-years^†^	0.011 (0.000–0.250)	0.003 (0.000–0.300)	0.011 (0.000–0.250)	0.378
*Lipids*				
Total cholesterol (mg/dl)	158.0 (139.0–179.0)	145.0 (129.5–164.5)	158.0 (140.0–178.8)	0.002
HDL cholesterol (mg/dl)	56.0 (48.0–66.0)	52.0 (45.0–62.5)	56.5 (48.0–66.0)	0.026
LDL cholesterol (mg/dl)	94.0 (78.0–111.0)	81.0 (69.0–103.0)	94.0 (78.0–111.0)	0.003
Triglycerides (mg/dl)	79.0 (59.0–106.0)	76.0 (58.0–111.0)	78.0 (60.0–106.0)	0.457
*Liver and inflammation markers*				
Alanine transaminase (U/l)	17.0 (13.0–22.0)	17.0 (15.0–23.5)	17.0 (13.0–22.0)	0.108
C-reactive protein (mg/dl)	0.15 (0.09–0.32)	0.15 (0.12–0.31)	0.16 (0.09–0.33)	0.572
*Carotid intima-media thickness*				
cIMT_MEAN_ (µm)	413.3 (381.7–446.7)	430.8 (399.6–463.8)	411.7 (380.0–445.0)	0.002

Values are given as number (%), mean ± standard deviation or median (Q_25_–Q_75_)

*p* Value comparison allergic asthma vs. subjects without any asthma or inhalative allergies using the Welch-t-test or the Chi-Square test as applicable

*BMI* body mass index, *HDL* high-density lipoprotein, *LDL* low-density lipoprotein, *cIMT*_*MEAN*_ mean carotid intima-media thickness

^†^Never smokers excluded

22 participants (representing 1.5% of all participants with a complete dataset) reported a physician diagnosis of asthma without an allergic component (non-allergic asthma). Among those, 16 (72.7%) subjects received anti-asthmatic medication. In both models, univariable and multivariable, participants with non-allergic asthma did not have significantly higher cIMT_MEAN_ compared to those without (411.7 vs. 411.7 µm; *p* = 0.932, adjusted R^2^ = 0.11) (Table 2). In participants with non-allergic asthma median cIMT_MEAN_ was 411.7 µm (388.8–439.6) [males: 438.3 µm (411.7–456.7), females: 400.0 µm (365.0–433.3); univariable *p* = 0.011].

**Table 2:** Association between asthma, inhalative allergy, allergic asthma and cIMT_MEAN_

Variable	Median cIMT_MEAN_ (µm)	Interquartile range (Q_25_–Q_75_)of cIMT_MEAN_ (µm)	Univariable *p* value	Multivariable *p* value
Physician diagnosed non-allergic asthma (*N* = 22)	411.7	388.8–439.6	0.932	0.953
Physician diagnosed inhalative allergy (*N* = 268)	420.0	385.0–449.6	0.118	0.925
Physician diagnosed allergic asthma (*N* = 58)	430.8	399.6–463.8	0.004	0.026

Multivariable linear regression included age, sex, size, waist circumference, systolic blood pressure, physical activity, cigarette pack years, low-density lipoprotein cholesterol, alanine aminotransferase, and C-reactive protein

268 participants (representing 19.0% of all participants with a complete dataset) had a physician diagnosis of inhalative allergy confirmed by a positive RAST and/or skin prick-test. Anti-allergic medication was taken permanently by 14 (5.2%) and on demand by 131 (48.9%) subjects. Again, in both models, cIMT_MEAN_ was not significantly higher in those subjects with inhalative allergic disorders compared to those without (420.0 vs. 411.7 µm; *p* = 0.118, adjusted R^2^ = 0.12) (Table 2). Median cIMT_MEAN_ in this subgroup was 420.0 µm (385.0–449.6) [males: 428.3 µm (401.7–466.7), females: 406.7 µm (375.8–430.0); univariable *p* < 0.001].

58 participants (representing 3.9% of all participants with a complete dataset) had a physician diagnosis of allergic asthma (i.e., simultaneous diagnosis of asthma and inhalative allergy). Among those, 56 (96.6%) subjects received anti-allergic and/or anti-asthmatic medication. Median cIMT_MEAN_ was 430.8 µm (399.6–463.8) [males: 445.8 µm (401.3–477.9), females: 416.7 µm (397.1–444.2); univariable *p* = 0.091] As primary outcome parameter, cIMT_MEAN_ was significantly higher in subjects with allergic asthma compared to those without (430.8 vs. 411.7 µm; univariable *p* = 0.004) and this association remained significant (multivariable *p* = 0.026, adjusted R^2^ = 0.12) after adjustment for established CVRF including age, sex, size, waist circumference, systolic blood pressure, physical activity, cigarette pack years, LDL-C, ALT, and CRP (Table 2). If only variables are included in the multivariable model, that significantly differ in the univariable analysis between participants with allergic asthma and those without asthma or inhalative allergies (sex, systolic blood pressure, physical activity, LDL-C, current smoker) or if waist circumference is replaced by weight, the significance level does not change significantly (*p* = 0.039 adjusted R^2^ = 0.07 or *p* = 0.027, adjusted R^2^ = 0.09).

## Discussion

In our large EVA-Tyrol cohort of healthy young adolescents, we could demonstrate an association between cIMT and allergic asthma, but not inhalative allergy or non-allergic asthma. A possible link between asthma and atherosclerosis has been a matter of debate for three decades. Several previous studies have demonstrated an association between asthma and an increased risk of CVD in adults [3–10]. However, the strength of this association varies widely and some of the results are limited to specific subgroups such as smokers [38] or women [39–42]. In addition, there are also studies that show no association between the two conditions [11, 12].

So far, only few studies have investigated the association between asthma and cIMT in pediatric populations. Cakmak et al. demonstrated that children with mild asthma had thicker cIMT than those without, but these results were not adjusted for potential confounders [25]. The Swiss Study on Air Pollution and Lung and Heart Disease In Adults (SAPALDIA)-Youth study came to a similar conclusion, although an increased cIMT was demonstrated for adolescent boys but not girls with asthma [27]. We have previously shown that in our cohort male sex is independently associated with an increased cIMT [37]. Even though there is a male preponderance in participants with allergic asthma, our finding remains robust when adjusting for potential confounders including sex.

Data on the effect of an allergic component on cIMT in asthmatic disease are even scarcer. To the best of our knowledge, only one small case–control study has previously investigated the association of allergic asthma and asthma in general with cIMT in children. In a modest sample size of 89 adolescents, increased cIMT was demonstrated in asthmatics with and without an allergic component compared to a healthy control group. In addition, asthmatics with an allergic component tended to have higher cIMT compared to those without, which fits well to our findings [43].

Based on these results and a previous study, which demonstrated an association of persistent asthma, but not intermittent asthma, with CVD events [4], it is tempting to speculate that due to high cumulative exposure to inhalative allergens and the resulting inflammatory processes, atherosclerotic vascular changes are detectable earlier in asthmatics with a strong allergic component than in those without.

A link between allergic asthma and atherosclerosis is not surprising. Atherosclerosis itself has been long recognized as an inflammatory process [13, 14] and chronic inflammatory diseases [15, 44] as well as autoimmune processes [14, 45] are linked to atherosclerosis and CVD. Regarding the pathophysiological background, three possible pathways could be discussed.

At the level of inflammatory mediators, the arachidonic acid metabolism is important because it can be associated with asthma as well as CVD. Polymorphisms in the 5-lipoxygenase-activating protein have been demonstrated to be associated with coronary heart disease [46] and 5-lipoxygenase inhibition showed protective effects in animal models of myocardial infarction [47] and atherosclerosis [48]. Mutations with decreased function in the human Phospholipases A2 gene—another key enzyme in the arachidonic acid metabolism—have been linked to atherosclerosis, coronary heart disease, stroke as well as asthma [49].

Another mechanism triggered mainly by inflammatory mediators is smooth-muscle remodeling. Hyperplasia and abnormal contraction of smooth muscle cells leading to airway obstruction in asthma are also recognized as features of vascular remodeling and endothelial abnormalities [50]. This pathophysiological mechanism may also explain the elevated blood pressure levels in allergic asthmatics compared to healthy controls in our study population. Previous studies have already reported an increased incidence of hypertension in adults with asthma or inhalative allergies [51, 52]. However, this association was not observed in all studies, particularly those with pediatric study cohorts [43, 53]. In our analysis, the association between allergic asthma and cIMT_MEAN_ was independent of blood pressure levels, making it unlikely that blood pressure is the sole link between asthma and cIMT_MEAN._

On a cellular level, the presence of the same sets of inflammatory cells in both the asthmatic bronchoalveolar and atherosclerotic vessel wall suggests that inflammatory cells share similar activities in both diseases [54]. Possible key roles play Immunoglobulin E (IgE)-triggered mast cells [55] and eosinophil leucocytes [56]. Through the release of chemokines and cytokines, both groups of cells elicit vascular permeability and increase the entry of LDL and inflammatory cells into the arterial wall fostering foam cell accumulation and atherosclerosis [55, 56].

Considering the described inflammatory links between asthma and atherosclerosis, the possibility arises that anti-inflammatory drugs could control both asthma and its disadvantageous effect on the vasculature. Previous small scale pharmacotherapeutic studies have already shown the potential benefit of various drug-based therapies, including inhaled corticosteroids [57, 58], lipid lowering statins [59], and 5-lipoxygenase inhibitor [60], yet sufficient powered randomized-controlled trials are lacking.

The strengths of our study include the large, homogeneous study cohort and a physician guided diagnosis of asthma from a face-to-face interview, as well as the requirement of diagnostic confirmation of the inhalative allergic component by a positive RAST and/or a prick-test. Still the absolute number of participants with allergic asthma was low, not allowing to further explore subgroups with different anti-allergic or anti-asthmatic treatment. Further limitations are the lack of determination of the duration and severity of asthma as well as the lack of determination of total and specific IgE-levels as markers for the degree of atopic sensitization.

## Conclusions

In summary, our analysis revealed significant associations between allergic asthma and increased cIMT in adolescents. Physicians should therefore be aware of allergic asthma as a potential CVRF in children and adolescents. The role of early and consequent anti-inflammatory treatment in prevention of CVD is unclear and merits further studies.

## Data Availability

The datasets used and/or analyzed during the current study are available from the corresponding author on reasonable request after appropriate ethics vote and signing of a data transfer agreement.

## Abbreviations

ALT: Alanine aminotransferase
ARFY: Atherosclerosis risk factors in female youngsters
ARMY: Atherosclerosis risk factors in male youngsters
BMI: Body-mass index
cIMT: Carotid intima-media thickness
cIMT_MEAN_: Mean carotid intima-media thickness
CRP: C-reactive protein
CVD: Cardiovascular disease
CVRF: Cardiovascular risk factor
EVA: Early Vascular Ageing
HDL-C: High-density lipoprotein cholesterol
IgE: Immunoglobulin E
LDL-C: Low-density lipoprotein cholesterol
RAST: Radio-allergo-sorbent-test
